# The Bayesian Susceptible-Exposed-Infected-Recovered model for the outbreak of COVID-19 on the Diamond Princess Cruise Ship

**DOI:** 10.1007/s00477-020-01968-w

**Published:** 2021-01-26

**Authors:** Chao-Chih Lai, Chen-Yang Hsu, Hsiao-Hsuan Jen, Amy Ming-Fang Yen, Chang-Chuan Chan, Hsiu-Hsi Chen

**Affiliations:** 1 Emergency Department of Taipei City Hospital, Ren-Ai Branch, Taipei, 106 Taiwan; 2 Institute of Epidemiology and Preventive Medicine, College of Public Health, National Taiwan University, Room 533, No. 17, Xu-Zhou Road, Taipei, 100 Taiwan; 3 Master of Public Health Program, National Taiwan University, Taipei, 100 Taiwan; 4 School of Oral Hygiene, College of Oral Medicine, Taipei Medical University, Taipei, 110 Taiwan; 5 Institute of Environmental and Occupational Health Science, College of Public Health, National Taiwan University, Taipei, 100 Taiwan; 6 Innovation and Policy Center for Population Health and Sustainable Environment, College of Public Health, National Taiwan University, Taipei, 100 Taiwan

**Keywords:** Bayesian SEIR model, COVID-19 outbreak, Diamond princess cruise ship, Prediction

## Abstract

The outbreak of COVID-19 on the Diamond Princess Cruise Ship provides an unprecedented opportunity to estimate its original transmissibility with basic reproductive number (R_0_) and the effectiveness of containment measures. We developed an ordinary differential equation-based Susceptible-Exposed-Infected-Recovery (SEIR) model with Bayesian underpinning to estimate the main parameter of R_0_ determined by transmission coefficients, incubation period, and the recovery rate. Bayesian Markov Chain Monte Carlo (MCMC) estimation method was used to tackle the parameters of uncertainty resulting from the outbreak of COVID-19 given a small cohort of the cruise ship. The extended stratified SEIR model was also proposed to elucidate the heterogeneity of transmission route by the level of deck with passengers and crews. With the application of the overall model, R_0_ was estimated as high as 5.70 (95% credible interval: 4.23–7.79). The entire epidemic period without containment measurements was approximately 47 days and reached the peak one month later after the index case. The partial containment measure reduced 63% (95% credible interval: 60–66%) infected passengers. With the deck-specific SEIR model, the heterogeneity of R_0_ estimates by each deck was noted. The estimated R_0_ figures were 5.18 for passengers (5–14 deck), mainly from the within-deck transmission, and 2.46 for crews (2–4 deck), mainly from the between-deck transmission. Modelling the dynamic of COVID-19 on the cruise ship not only provides an insight into timely evacuation and early isolation and quarantine but also elucidates the relative contributions of different transmission modes on the cruise ship though the deck-stratified SEIR model.

**Supplementary information:**

The online version contains supplementary material available at (10.1007/s00477-020-01968-w)

## Introduction

The ongoing pandemic of coronavirus disease 2019 (COVID-19), caused by severe acute respiratory syndrome coronavirus 2 (SARS-CoV-2), was first identified in Wuhan, Hubei, China in December 2019 (WHO 2020; Huang et al. 2020; Guan et al. 2020; Li et al. 2020), and then declared as a Public Health Emergency of International Concern (PHEIC) on January 30th, 2020 and a global pandemic on March 11st, 2020 (WHO 2020). To contain such rapid and wide spread of COVID-19 outbreak, it is imperative to unbiasedly estimate the basic reproductive number (R_0_) defined as the average number of secondary cases generated from a primary case, a useful indicator of infectious disease transmission, locally and globally. One can use R_0_ larger or smaller than 1, determined by the contact rate, transmission probability, and infectious period, to assess the possibility of potential spread or the control of COVID-19 in affected communities (Ferguson et al. 2005).

At the beginning of COVID-19 epidemic, several studies have tried to quantify R_0_ by the models based on parameters derived from the SARS epidemic in 2003 (Wu et al. 2020; Imai et al. 2020a, b), which was estimated as 2–3, a value close to the R_0_ of SARS (Raily et al. 2003). As the epidemic evolves with time, the difference between SARS and COVID-19 has been gradually noted in terms of transmission mode and infectious period in relation to clinical symptoms and signs (Huang et al. 2020; Guan et al. 2020; Li et al. 2020; Wang et al. 2020). The properties of asymptomatic infection and various clinical symptoms and signs of COVID-19 render the estimation of the rate of spread with the SARS models intractable. The disparity of transmission between SARS and COVID-19 has been further speculated by the rampant spread in the Washington and the New York states of USA with a double increase from March 26th to April 1st and also in the beginning epidemics of European countries such as Italy and Spain (WHO 2020).

The COVID-19 outbreak on the Diamond Princess Cruise Ship (NIID 2020a, b) provides an unprecedented opportunity for us to understand its original transmissibility and force of spread by four unique epidemic-related characteristics. First, susceptible, contact, transmission, and infectious period are clearly defined according to a fixed cohort of global passengers and crews living at different levels of deck in the isolated setting of this cruise ship. Second, such a transmission mode through the isolated environment of the cruise ship provides strong human-to-human community-acquired infection in close setting distinct from the transmission mode through household or hospital and relevant healthcare institutions while assessing the transmissibility of this emerging infectious disease. The third is that the infected persons on board consist of multi-nationals giving us a better sense of the transmissibility of COVID-19 across racial groups. The fourth is that modelling the heterogeneity of transmission by different levels of decks with passengers and crews also give a clue to relative contribution between different transmission modes such as aerosol and fomite transmission based on assessment of whether the main route of transmission through the within-deck transmission or the between-deck transmission. Based on these four characteristics related to the transmission mode on the cruise ship, three thorny questions that have been not addressed in the previous studies are raised (Mizumoto et al. 2020; Rocklöv et al. 2020; Sawano et al. 2020). Can we estimate and predict the epidemic curve to determine timely containment measures and evacuation? What is the effectiveness of containment measures on quarantine and isolation for the infected passengers before evacuation? What is the main route of transmission responsible for the outbreak of COVID-19 on the cruise ship? Answering these three questions plays a crucial role in the guidance of how and when containment measures can be designed and offered for the coming cruise ships that are susceptible to the outbreak of COVID-19. Answering these three questions requires the development of a mathematical modelling for estimating basic reproductive number in relation to the outbreak of COVID-19 on cruise ship.

Although the conventional deterministic approach by fitting the compartment model with the ordinal differential equation (ODE) system to the observed number of reported cases can shed light on the dynamic of COVID-19 transmission and R_0_ on population level (Lyra et al. 2020), such an approach may not be appropriate for assessing the outbreak occurring on the cruise ship with modest size of susceptibles particularly when the uncertainty of parameters needs to be considered. As far as the conventional maximum likelihood estimate (MLE) method is concerned, it may involve the identifiability problem while estimating multiple parameters such as transmission coefficient, recovery rate, and incubation period in this study. The conditional approach is therefore required to estimate the parameter of interest such as transmission coefficient given the already known and stable estimates like recovery rate and incubation period with information borrowing form literatures. Thus, the uncertainty of the estimated parameter may not be precise. Here, we first developed a Bayesian compartment model for the COVID-19 outbreak on the Diamond Princess Cruise Ship. To cope with the uncertainty of full joint parameters related to the outbreak in such a confined environment, we then applied the Bayesian Markov Chain Monte Carlo (MCMC) estimation method, in contrast to both deterministic and maximum likelihood estimate (MLE) methods, to estimate the transmission coefficient, recovery, and incubation period, and their 95% credible intervals (CrI) of COVID-19 outbreak on the Diamond Princess Cruise Ship.

The rest of the article is organized as follows. Section 2 briefs the empirical data on the outbreak of COVID-19 on the Diamond Princess Cruise Ship alone with the events took place in the quarantine measures on board. Section 3.1 gives the details on the specification of the ODE compartment models with Bayesian underpinning to depict the propagation of COVID-19 through the states of susceptibles, exposed, infected, and removed (recovery and death). Section 3.2 extended the Bayesian compartment equation for each deck to account for the heterogeneity of SARS-CoV-2 transmission on the Diamond Princess Cruise Ship. Section 3.3 provides three estimation methods, including Bayesian MCMC method, MLE, and deterministic approach. The methods for validating the fitted cases, predicting dynamic epidemic curves on the evolution of susceptibles, exposed, infected, and recovery, and evaluating the efficacy of containment measures are addressed in Sect. 3.4. Section 4.1 shows the estimated results on the transmissibility of SARS-CoV-2 with three estimation methods. Section 4.2 provides the dynamic of COVID-19 outbreak on the Diamond Princess Cruise Ship. Section 4.3 shows the heterogeneity of COVID-19 transmission on the Diamond Princess Cruise Ship by the deck-stratified SEIR model. Section 4.4 shows the efficacy of containment measures. Section 5 gives the discussion of applications and implications on the proposed Bayesian ODE compartment models to assess the disease outbreak data.

## Empirical data

### Data on COVID-19 outbreak of the Diamond Princess cruise ship

The data on the voyage of Diamond Princess Cruise Ship, the reported number of confirmed COVID-19 cases, and the quarantine process were retrieved from publicly available sources including the Princess Cruise website of the Carnival Cooperation (Princess Cruise Lines 2020), the official website of Ministry of Health, Labor and Welfare, Japan (Ministry of Health, Labor and Welfare, Japan 2020), and the National Institute of Infectious Disease, Japan (NIID 2020a, b). The auxiliary information sources including the general media, scientific news, and web-based summaries were also used for a cross-check on the cases numbers and the quarantine measures and the evacuation after the outbreak on the Diamond Princess Cruise Ship (Princess Cruise Lines 2020; Wikipedia 2020a, b; Garcia-Navarro 2020; Normile 2020). A prospective observational study design on the propagation of the COVID-19 outbreak was applied to evaluating the transmissibility of SARS-CoV-2. Details on the derivation of information on COVID-19 outbreak on the Diamond Princess Cruise Ship are provided in the Supplementary material A.

### COVID-19 outbreak on the Diamond Princess Cruise Ship

The Diamond Princess Cruise Ship departed from Yokohama on January 20^th^, 2020. There were a total of 3711 subjects including 2666 passengers from 11 countries (1285 from Japan, 470 from Hong Kong, 425 from USA, 215 from Canada, 40 from United Kingdom, 25 from Russia, 20 from Taiwan, 15 from Israel, 13 from New Zealand, and the rest of 158 from Australia and Germany) and 1045 crew members on the voyage of the Diamond Princess (NIID 2020a; Ministry of Health, Labor and Welfare, Japan 2020; Wikipedia 2020a). The outbreak started from a passenger from Hong Kong who had joined with part of the voyage and disembarked on January 25th, 2020 who was later confirmed as a COVID-19 case through laboratory testing (Ministry of Health, Labor and Welfare, Japan 2020; Wikipedia 2020a). The Diamond Princess Cruise Ship was therefore quarantined at the Yokohama harbor since its dock on February 5th, 2020. Following the index case, 10 cases of COVID-19 were reported on February 5th, 2020, which heralded the outbreak yielding 762 cases in the following weeks (WHO 2020; Ministry of Health, Labor and Welfare, Japan 2020; Wikipedia 2020a). Up to February 28th, the COVID-19 outbreak on the Diamond Princess Cruis Ship have resulted in 6 deaths (WHO 2020).

The heterogeneity in SARS-CoV-2 transmission by deck was evaluated by using the data on total number of COVID-19 cases up to February 13th and the summarized information retrieved from data on the distribution of COVID-19 cases among passengers and crews onboard for each deck in the early period of COVID-19 outbreak on the Diamond Princess Cruise Ship published by Yamagishi et al. (2020a, b).

### Containment measures for COVID-19 transmission on the Diamond Princess Cruise Ship

During the quarantine process, the personal protective equipement was provided for crew members. Passangers and crews were instructed to monitor body temperature by themselves. Subjecs with body temperature higher than 37.5 degree Celsius or developed any illness were referred to the medical center on the ship where medical services were provided and the test for SARS-CoV-2 were referred. To maintain operations of the ship, some crew continued to perform essential but limited services while the ship remained in quarantine (NIID 2020a). This symptom-based approach started since the ship dock at Yokohama harbored on February 5th. With the gradual expansion of laboratory capacity from February 11st, the quarantine officers provided test systematically gauged by testing capacity and covered passengers without the manifestation of symptoms from February 14th (NIID 2020a, b).

**Fig. 1:**
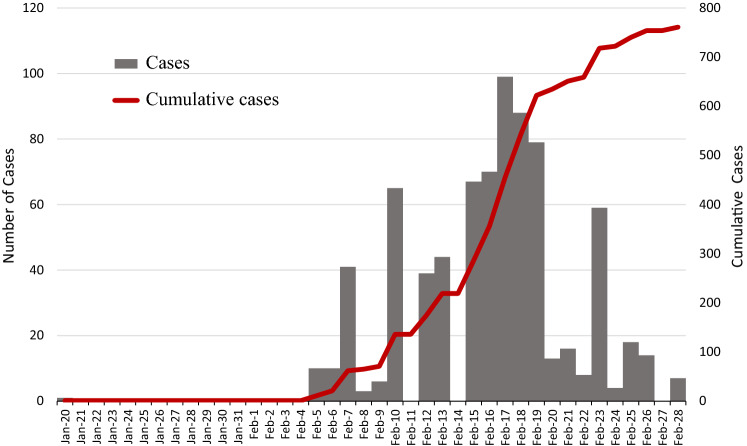
The epidemic curve and cumulative cases of COVID-19 on the Diamond Princess Cruise Ship. The daily reported COVID-19 cases (gray bar, left Y-axis) and cumulative cases (red line, right Y-axis) on the Diamond Princess Cruise Ship from January 20th to February 28th, 2020

**Figure ****1** shows both the epidemic curve and cumulative cases of all passengers on the Diamond Princess Cruise Ship during January 20th–February 28th, 2020. The cases were identified mainly on the basis of the symptom of fever and the close contacts defined by sharing the cabin with confirmed cases during the quarantine process followed by laboratory test (NIID 2020a, b; Ministry of Health, Labor and Welfare, Japan 2020). Passengers who were tested positive for SARS-CoV-2 were removed from the ship and transferred to health care facilities on land. Subjects with negative test result will be kept in the ship for the rest of quarantine period. The COVID-19 cases identified on the Diamond Princess Cruise Ship were reported on daily basis by the Ministry of Health, Labor and Welfare, Japan (2020). On February 16th, 2020, several countries started requesting the evacuation of their citizens from the cruise ship. The USA was the first country to pull out 380 Americans on February 17th, 2020. It should be noted that the quarantine measure has commenced from February 5th but isolation and decontamination were gradually expanded from Feb 14th until the complete evacuation on February 19th, 2020 (NIID 2020a, b).

The data used for the following analysis consisted of the cases reported between January 20th to February 19th, 2020, the date of ending quarantine announced by Ministry of Health, Labor and Welfare, Japan. Detailed counts of the cases and the information sources are provided in Supplementary Material S-Table 1. The positive rate for SARS-CoV-2 among passengers tested was 20.6% (621/3011). Among 621 SARS-CoV-2 infected cases, 322 were asymptomatic (51.9%, S-Table 1 in the Supplementary Material).

S-Table 2 summarizes the frequencies of passengers (S-Table 2 (a)) and crews (S-Table 2(b)) by decks. Up to Feb 13th, 194 (7.3%) and 25 (2.4%) COVID-19 cases were identified among the 2666 passengers and 1045 crews, respectively. The detailed information on susceptible and infected COIVID-19 cases of each deck is also listed in S-Table 2.

## Bayesian SEIR model for the outbreak of COVID-19

### Model specification

**Fig. 2:**
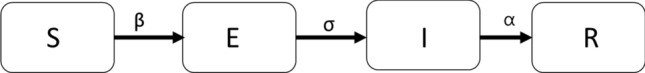
The SEIR (Susceptible-Exposed-Infected-Recovery) model for the evolution of COVID-19 on the Diamond Princess Cruise Ship

To predict the propagation of COVID-19 outbreak on the cruise ship with a better use of the observed number of overall and deck-specific cases, making allowance for the uncertainty of parameters, we applied the ordinary differential equations (ODE) to capture dynamics of the SEIR model at instantaneous time with Bayesian underpinning. A four-compartment model (Fig. 2) was diagrammed to delineate the daily flow of the population on the Diamond Princess Cruise Ship with respect to COVID-19 infection, including S (susceptible population), E (exposed to infectives), I (infected with COVID-19), and R (recovered from infectious status or infected persons under isolation or removed from the cruise ship, or dead) states.

Let *s*(*t*), *e*(*t*), *i*(*t*), and *r*(*t*) denote the numbers of susceptibles, exposed, infected, and removed/isolated/recovered/dead on the cruise ship at time *t*. The four-compartment system can be depicted by using the following ordinary differential equations (ODEs) to describe the instantaneous change of the four states with time

1 $$\begin{aligned} \frac{{ds\left( t \right)}}{{dt}} & = - \frac{{\beta \;s\left( t \right)\;i\left( t \right)}}{N} \\ \frac{{de\left( t \right)}}{{dt}} & = \frac{{\beta \;s\left( t \right)\;i\left( t \right)}}{N} - \sigma \;e\left( t \right) \\ \frac{{di\left( t \right)}}{{dt}} & = \sigma \;e\left( t \right) - \alpha \;i\left( t \right) \\ \frac{{dr\left( t \right)}}{{dt}} & = \alpha \;i\left( t \right) \\ \end{aligned}$$

Following the Eq. (1), the cumulative frequency for each state up to time *t* can be expressed as:

2 $$\begin{aligned} s\left( t \right) & = \mathop \smallint \limits_{0}^{t} - \frac{{\beta \;s\left( u \right)\;i\left( u \right)}}{N}du \\ e\left( t \right) & = \mathop \smallint \limits_{0}^{t} \frac{{\beta \;s\left( u \right)\;i\left( u \right)}}{N} - \sigma \;e\left( u \right)du \\ i\left( t \right) & = \mathop \smallint \limits_{0}^{t} \sigma \;e\left( u \right) - \alpha \;i\left( u \right)du \\ r\left( t \right) & = \mathop \smallint \limits_{0}^{t} \alpha \;i\left( u \right)du~ \\ \end{aligned}$$

Solving the system of differential Eq. (2) is subject to the initial values specified by s(0) = 3710, e(0) = 0, i(0) = 1, and r(0) = 0, respectively. With the solution of the four-state differential equations regarding the empirical data on the observed COVID-19 cases on the Diamond Princess Cruise Ship, the parameters of transmission coefficient (*β*), average incubation period (1/*σ*), and recovery rate (*α*) from the infectious status can be estimated. The estimated results were further used to depict the propagation on the number of four COVID-19 infective states by time. Note that the SEIR model is a variant of the SIR model with a further consideration of a significant incubation period during which individuals have been infected but are not yet infectious themselves and stay in compartment E (Wikipedia 2020c).

Note that because the fixed population of the Diamond Princess Cruise Ship, the following equation always holds up to the end of quarantine on February 19th.

3 $$s\left( t \right) + e\left( t \right) + i\left( t \right) + r\left( t \right) = N$$

where *N* is the initial size of this cohort, namely a total of 3711 subjects on the Diamond Princess Cruise Ship.

### The SEIR model for the deck-specific dynamic of COVID-19

The proposed ODE Eq. (1) for the SEIR model was further refined by using a vector notation with the capital symbol to capture the heterogeneity of SARS-CoV-2 transmission in the stratified passengers or crew for each deck following the stratified method proposed by Daley and Gani (2001). Suppose there are *k* decks on the Diamond Princess Cruise Ship, let **S**(t), **E**(t), **I**(t), and **R**(t) denote a series of *j* (= 1,2,3,…,*k*) column vectors at time t representing the number of suseceptibles, exposed, infected, and removed/isolated/recovered/dead, respectively. The column vectors for **S**(t), **E**(t), **I**(t), and **R**(t) are specified by

$$S\left( t \right) = \left[ {\begin{array}{*{20}c} {s_{1} \left( t \right)} \\ {s_{2} \left( t \right)} \\ \vdots \\ {s_{k} \left( t \right)} \\ \end{array} } \right]E\left( t \right) = \left[ {\begin{array}{*{20}c} {e_{1} \left( t \right)} \\ {e_{2} \left( t \right)} \\ \vdots \\ {e_{k} \left( t \right)} \\ \end{array} } \right] ,I\left( t \right) = \left[ {\begin{array}{*{20}c} {i_{1} \left( t \right)} \\ {i_{2} \left( t \right)} \\ \vdots \\ {i_{k} \left( t \right)} \\ \end{array} } \right], \,and\,R\left( t \right) = \left[ {\begin{array}{*{20}c} {r_{1} \left( t \right)} \\ {r_{2} \left( t \right)} \\ \vdots \\ {r_{k} \left( t \right)} \\ \end{array} } \right]$$

Note that the total number of subjects as in the Eq. (3), *s*_*j*_(t) + *e*_*j*_(t) + i_*j*_(t) + *r*_*j*_(t) = N_*j*_ holds for each stratum *j*. The corresponding ODE equation for the deck-specific population is expressed as:

4 $$\begin{gathered} \frac{dS\left( t \right)}{{dt}} = - S\left( t \right) \times \left( {I^{T} \left( t \right) \times B} \right) \hfill \\ \frac{dS\left( t \right)}{{dt}} = - S\left( t \right) \times \left( {I^{T} \left( t \right) \times B} \right) \hfill \\ \frac{dI\left( t \right)}{{dt}} = \sigma \times E\left( t \right) - \alpha \times I\left( t \right) \hfill \\ \frac{dR\left( t \right)}{{dt}} = \alpha \times I\left( t \right) \hfill \\ \end{gathered}$$

where **I**^T^(t) represents the transposed vector of **I**(t). The corresponding *k* × *k* matrix of transmission coefficients, **B**, is expressed as

5 $$B = \left[ {\begin{array}{*{20}c} {\beta_{11} /N_{1} } & {\beta_{12} /N_{1} } & \cdots & {\beta_{1k} /N_{1} } \\ {\beta_{21} /N_{2} } & {\beta_{22} /N_{2} } & \cdots & {\beta_{2k} /N_{2} } \\ \vdots & \vdots & \ddots & \vdots \\ {\beta_{k1} /N_{k} } & {\beta_{k2} //N_{k} } & \cdots & {\beta_{kk} /N_{k} } \\ \end{array} } \right]$$

where β_*jl*_ captures the between-deck transmission coefficients of SRAS-CoV-2 between stratum *j* and stratum *l* by using the off-diagonal elements of **B** with *j* ≠ *l*. The diagonal elements of **B**, *β*_*jj*_, represent the within-deck transmission for *j* = *l.*

### Estimation of parameters

#### Bayesian MCMC estimation method

Given the proposed ODE-based SEIR model, the Bayesian MCMC method was used to estimate the parameters of interests. The Bayesian approach facilitates us to incorporate the prior information of parameters. As COVID-19 is an emerging disease, little is known about the time interval between exposed and infected, as well as between infected and recovery. We borrowed the experience from the first report in Wuhan that is the origin infectious resource for the outbreak of the Diamond Princess Cruise Ship. We then assigned Gamma (53, 278) and Gamma(24, 168) for $\sigma$ and $\alpha$ in order to fit the mean duration from exposed to infective and from infection to recovery for 5.25 (95% CrI: 4–7) and 7 (95% CrI: 5–12) days, respectively (Huang et al. 2020; Guan et al. 2020).

Since only data on the number of reported confirmed cases per day were available, they contain those already infected, regardless of appearance of clinical symptoms and signs, and recovered cases, we therefore modeled two random variables, number of unknown infected status and the confirmed cases per day following two normal distributions of

6 $$Normal\left( {s\left( t \right) + e\left( t \right), 100} \right)\;{\text{and}}\;Normal\left( {i\left( t \right) + r\left( t \right), 100} \right)$$

respectively.

Following the derivation of transmission coefficient, *β*, the basic reproductive number (R_0_) was estimated with function of transmission coefficient (*β*) and recovery rate (*α*) as follows,

7 $$R_{0} = \beta /\alpha$$

Regarding the proposed deck-specific SEIR model by deck, we first estimated the within-deck transmission coefficients (β_*jj*_) and the corresponding basic reproductive number estimates by using the information on the frequencies of the COVID-19 cases for passengers and crews on each deck. Due to the sparse data stratified by deck during the early phase of outbreak, a Poisson distribution indicated by the expected number of cases and recovered for each deck, (*i*_*j*_(t) + *r*_*j*_(t)) was applied to estimating β_*jj*_*.* The Eq. (6) was thus written by

8 $$Poisson\left( {i_{j} \left( t \right) + r_{i} \left( t \right)} \right)$$

The within-deck basic reproductive number was derived by *β*_*jj*_/α based on the Eq. (7).

The expected COVID-19 cases resulting from within-deck transmission can be calculated from the estimated results of the diagonal elements *(β*_*jj*_*)* based on the Bayesian SEIR model for each deck. The proportion of COVID-19 cases in each deck attributed to between-deck transmission can be approximated by subtracting the expected within-transmission from the fixed total number of COVID-19 cases.

The Bayesian Markov Chain Monte Carlo (MCMC) method for the ordinary differential equation system for the four-compartment SEIR model was used to estimate parameters of interests. A series of SEIR models with Bayesian MCMC approach were applied to quantifying the force of COVID-19 spreading by using the data up to February 19th, 2020. S-Table 4 summarizes the data, estimated parameters, and posterior distribution of the Bayesian SEIR model.

#### Deterministic and maximum likelihood estimation methods

Here, we also make the comparisons of the estimated results using the abovementioned Bayesian MCMC estimation method with the corresponding estimated results using both deterministic and MLE estimation methods.

Based on the four-compartment ODE system specified by (1), the transmission coefficient, *β*, can be estimated from the empirical data on cumulative COVID-19 cases given the incubation time (the inverse of *σ*) and recovery rate (*α*) following the deterministic approach. The average value for the two parameters was thus abstracted from the first report in Wuhan (Huang et al. 2020; Guan et al. 2020). With these information, we thus applied the dwelling time of 5.25 days (σ = 0.19) and 7 days (α = 0.1428) for the compartment E and I, respectively.

We also applied the MLE method to estimating the R_0_ and *β* by using the observed data on the cumulative COVID-19 cases on Diamond Princess Cruise Ship. Following the SEIR model depicted by Eq. (1) and (2), the MLE for the parameters of SEIR model were derived by maximizing the likelihood function written as follows

9 $$L(\beta ,\sigma ,\alpha ) = \Pi_{i} [s(t_{i} ) + e(t_{i} )]^{{N_{{t_{i} }} }} \;[i(t_{i} ) + r(t_{i} )]^{{C_{{t_{i} }} }}$$

where *N*_*ti*_ and *C*_*ti*_ correspond to the observed data on the number of subjects with unknown infected status and the confirmed cases per day as the random variable modelled in the Bayesian approach of Eq. (4). To reduce the identifiability problem of estimating three parameters simultaneously, we estimated the main parameter of transmission coefficient given the fixed value of the inverse of *σ* and recovery rate (*α*) derived from those used in the deterministic approach mentioned above.

### Model validation, prediction of dynamics, and evaluation of effectiveness on containment measures on CIVID-19 outbreak on Diamond Princess Cruise Ship

To assess the homogeneity of estimated results on transmission coefficient and basic reproductive number derived from the Bayesian ODE-based SEIR compartment model across the quarantine period, data up to February 13th (before systemic test), February 16th (before the evacuation of passengers from USA), and February 19th were used. The validation of the model was assessed by comparing the fitted cumulative COVID-19 cases with the observed number using three estimation methods.

Moreover, in order to assess whether the confirmed-cases-per-day originated from exhaustive daily testing, which would confirm the appropriateness of combing susceptible and exposed versus infected and recovery given a substantial proportion of eventual cases turned out to be asymptomatic, we also compared the fitted cases with the observed ones on daily basis to check the early period from February 5th to 10th based on daily symptom-reported only and the subsequent period with daily testing.

Based on the estimated three parameters, the daily epidemic curves on the dynamics of COVID-19 can be also derived to show the evolution of susceptibles, exposed, infected, and recovery during the COVID-19 outbreak on the Diamond Princess Cruise Ship.

The efficacy on the quarantine measures with the consideration of evacuation was further assessed on the basis of the estimated result derived from the period between January 10th to February 19th in conjunction with the extended data after the evacuation to predict the cases up to February 28th, 2020. The evaluation of efficacy was derived by the comparison with the total observed cases up to February 28th including the index case from Hong Kong.

## Results

### Transmissibility of SARS-CoV-2 on cruise ship

By applying three estimation methods to daily reported cases series, the estimated results on the transmission coefficient and R_0_ for the COVID-19 outbreak on the Diamond Princess Cruise Ship are listed in Table 1. The R_0_ and transmission coefficient (β) was estimated as 5.27 and 0.75/day, respectively, based on the cases up to February 19th, 2020 by using the deterministic method. Note that the figure of 0.75/day is a reflection of transmission coefficient that takes into account the instantaneous change of the SEIR model as depicted in a series of differential Eqs. (2) in contrast the average rate of 0.2/day on COVID-19 occurrence up to Feb-19. This figure does not. To this end, we applied the Bayesian SEIR model to estimate the transmission coefficient taking into account the dynamic changes in the number of infectives, exposed, infected, and recovered. For the MLE method, the estimates of R_0_ and β was estimated as 5.43 and 0.78/day, respectively. The corresponding figures derived from the Bayesian ODE-based SEIR model were 5.70 (95% credible interval (CrI): 4.23–7.79) and 0.79/day (95% CrI: 0.64–0.97), respectively. The results on the point estimates of R_0_ and β were similar across three estimation methods.

**Table 1:** Estimated basic reproductive numbers of SARS-CoV-2

Scenario	Parameter	Deterministic approach	MLE for ODE SEIR model*	Bayesian MCMC for ODE SEIR model*
		Estimate	Estimate (95% CI)	Estimate (95% CrI)
Up to 2020-02-13	Basic reproductive number (R_0_)	5.70	5.39 (5.29, 5.50)	5.67 (4.09, 8.02)
	Transmission coefficient (β)	0.82	0.77 (0.76, 0.79)	0.79 (0.62, 1.02)
Up to 2020-02-16	Basic reproductive number (R_0_)	5.27	5.38 (5.29, 5.47)	5.66 (4.16, 7.82)
	Transmission coefficient (β)	0.75	0.77 (0.76, 0.78)	0.78 (0.63,0.98)
Up to 2020-02-19	Basic reproductive number (R_0_)	5.27	5.43 (5.43, 5.43)	5.70 (4.23, 7.79)
	Transmission coefficient (β)	0.75	0.78 (0.78, 0.78)	0.79 (0.64, 0.97)

*MLE maximum likelihood estimate; *MCMC* Markov Chain Monte Carlo, *SEIR model* Susceptible-Exposed-Infected-Recovery model; *CI* confidence interval; *CrI* credible interval

**Fig. 3:**
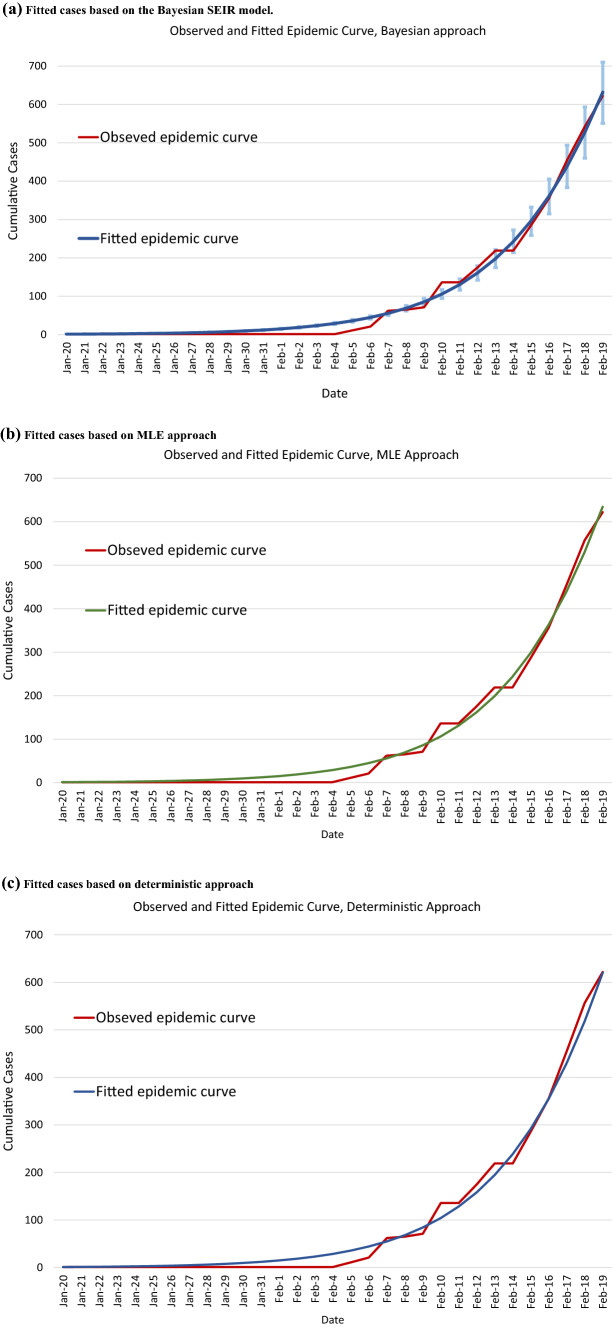
The fitted cases versus the observed cases for the COVID-19 outbreak on the Diamond Princess Cruise Ship. (**a**) Fitted cases based on the Bayesian SEIR model. (**b**) Fitted cases based on MLE approach. (**c**) Fitted cases based on deterministic approach

Consistent results were derived when different time points for the observed epidemic with the estimated R_0_ ranging between 5.66 to 5.70 with the overlapping of 95% CrIs. The Bayesian ODE-based SEIR model demonstrated a satisfactory fit by using the information on data prior to the February 19th, 2020 as shown in Fig. 3 and Table 2.

**Table 2:** Observed and expected cumulative COVID-19 cases by date using data up to designated dates

Date	Observed cumulative cases	Use data up to 2020-02-13		Use data up to 2020-02-16		Use data up to 2020-02-19	
		Expected cases		Expected cases		Expected cases	
		Average	95% CrI	Average	95% CrI	Average	95% CrI
Feb-5	11	35.8	(23.5, 46.9)	35.2	(29.3, 40.7)	35.9	(32.5, 39.0)
Feb-6	21	44.6	(28.5, 59.3)	43.7	(36.2, 51.0)	44.6	(40.2, 48.6)
Feb-7	62	55.5	(35.8, 76.0)	54.2	(44.5, 63.9)	55.4	(50.0, 60.8)
Feb-8	65	68.9	(43.5, 95.8)	67.2	(54.3, 79.4)	68.8	(61.8, 75.6)
Feb-9	71	85.5	(52.1, 119.9)	83.3	(66.8, 99.4)	85.3	(76.5, 94.3)
Feb-10	136	106.0	(64.2, 151.7)	103.0	(82.0, 124.0)	105.5	(94.3, 117.2)
Feb-11	136	131.1	(75.2, 187.6)	127.2	(100.0, 153.9)	130.4	(116.0, 145.1)
Feb-12	175	161.9	(90.1, 233.6)	156.7	(121.7, 190.6)	160.9	(142.0, 179.0)
Feb-13	219	199.3	(111.0, 292.7)	192.7	(148.0, 235.4)	198.0	(174.8, 221.5)
Feb-14	219	–	–	236.3	(181.7, 291.7)	242.9	(213.8, 272.6)
Feb-15	286	–	–	288.8	(221.0, 358.6)	297.0	(258.7, 332.1)
Feb-16	356	–	–	351.4	(267.8, 438.0)	361.7	(314.9, 405.4)
Feb-17	455	–	–	–	–	438.2	(383.0, 493.6)
Feb-18	543	–	–	–	–	527.9	(459.9, 593.0)
Feb-19	622	–	–	–	–	631.9	(551.0, 709.9)

Although point estimates were similar among three methods, the deterministic approach did not consider the uncertainty of three parameters. The MLE method only allowed for the uncertainty of transmission coefficient but did not consider recovery rate and incubation period because the identifiability problem were encountered while three parameters were jointly estimated by using the MLE method. Both two nuisance parameters (recovery rate and incubation period) were therefore conditioned as fixed values while the main parameter of transmission coefficient was estimated by the MLE method. By eliciting informative priors of recovery rate and incubation period, the Bayesian approach were able to yield three point estimates and also the corresponding 95% CrIs of three parameters. This accounts for lacking confidence interval (CI) for transmission coefficient (β) and basic reproductive number (R_0_) using the deterministic approach and also narrow CIs for both of them using the MLE method compared with the wider 95% CrI using the Bayesian MCMC method as the joint uncertainty of three parameters was considered.

Figure 3a shows the observed cases as opposed to the fitted ones on cumulative cases derived from the Bayesian ODE-based SEIR model with the detailed fitted cumulated cases listed in Table 2. Based on the transmission parameter estimated by the empirical data from January 20th to February 19th, the fitted cumulated cases on February 19th are 632 (95% CrI: 551–701), which is comparable to the observed cumulative cases of 622 (χ^2^_(1)_ = 0.16, *P* = 0.69). The graphic diagram with cumulative cases as shown in Fig. 3(a) not only provides a clear visualization with smoothing but also demonstrates 95% predicted credible interval for capturing the joint uncertainty of three parameters encoded in the posterior distribution with time. Figure 3b and c also shows the fitted curve of COVID-19 based on the results of MLE and deterministic approach.

In addition to comparing the cumulative fitted frequencies with the observed ones, in order to confirm the appropriateness of combining the components of suspected and exposed (*s*(*t*) + *e*(*t*)) and infected and recovered (*i*(*t*) + *r*(*t*)) used in the Bayesian ODE-based SEIR model, we also compared the predicted daily frequencies of COVID-19 cases with the observed ones (S-Table 3). While most of the cumulative fitted frequencies derived from the Bayesian ODE-based SEIR model were in line with the cumulative observed one (Fig. 3 and S-Table 5), the fitted daily frequencies were deviated from observed one during February 5th–11st because only a symptomatic-based case identification during this early period was adopted and infected cases would be accumulated until the presence of symptom and signs as seen on February 7th and 10th. After February 11st, the quarantine offices provided daily test systematically gauged by testing capacity and covered passengers without the manifestation of clinical symptoms. The fitted frequencies of COVID-19 cases between February 12nd and February 19th, after the expansion of testing capacity, were thus close to the observed one (S-Fig. 1 and S-Table 3). Such a finding supports the appropriateness of combining the components of suspected and exposed (*s*(*t*) + *e*(*t*)) and infected and recovered (*i*(*t*) + *r*(*t*)) used in the Bayesian ODE-based SEIR model.

### The dynamic of COVID-19 on cruise ship

**Fig. 4:**
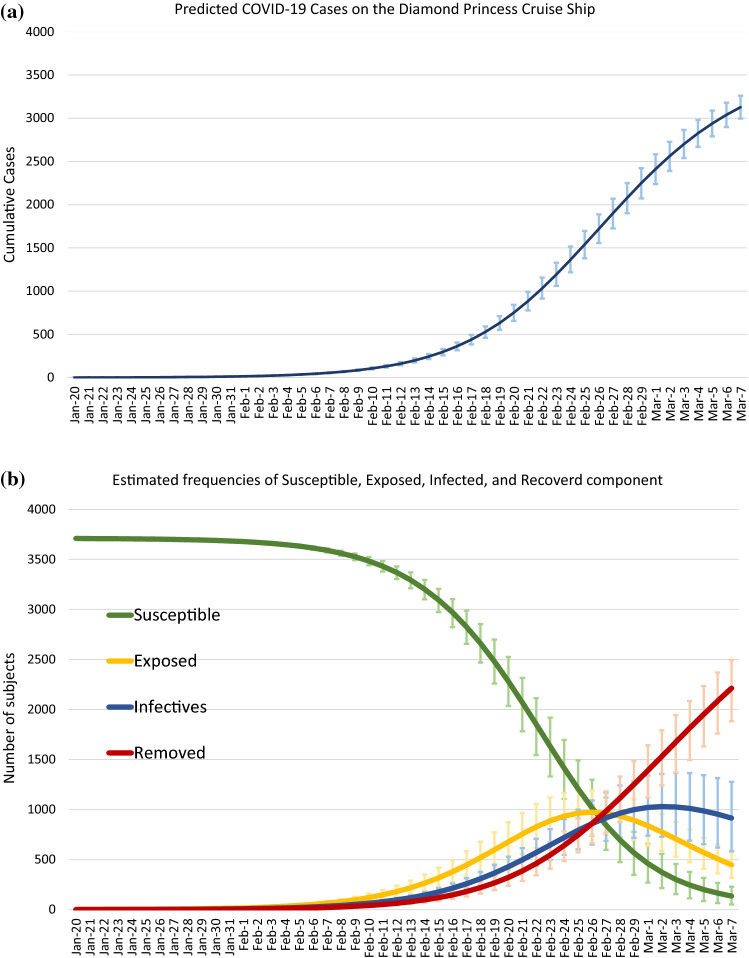
Predicted COVID-19 cases. (**a**) shows the predicted number of daily cumulated COVID-19 cases that occurred on the Diamond Princess Cruise Ship given the scenario of no containment measures up to Mar. 7th. (**b**) shows the dynamic curves of the SEIR model, including the frequencies of the components of susceptible (S, green line), exposed (E, yellow line), infected (I, blue line), and removed (R, red line) along with the estimated 95% credible intervals on the dynamics of each component

Based on the parameters of basic reproductive number trained by the data before February 19th, Fig. 4a shows the predicted cumulative curve given the scenario of no containment measures to control the COVID-19 outbreak on the Diamond Princess Cruise Ship with the detailed daily predicted counts listed in S-Table 5 in the Supplementary Material. While the total of 3128 (95% CrI: 2995–3261) were infected and only 16% passengers remain susceptible the outbreak would be expected to end on March 7th according to the theory of herd immunity when more than 82% [(1–1/5.70) × 100%] of total population have been infected. The evolution of COVID-19 outbreak can further be decomposed into the status of susceptible, exposed, infected, and removed (including recovery and death) among the 3711 passengers of the Diamond Princess Cruise Ship. Figure 4(b) shows this dynamic epidemic curve in terms of these four components. The passengers were exposed to an increasing risk of being infected by SARS-CoV-2 and further developed into COVID-19 during the period of January 20th to February 26th which reached the peak of 974 (95% CrI: 748–1193) on February 26th. On February 19th, 2020, the number of subjects with effective exposure and infected with SARS-CoV-2 without the manifestation of clinical symptom for COVID-19 was estimated as 598 (95% CrI 431–773) (S-Table 6 in the Supplementary Material), while the COVID-19 cases were predicted as 632 (95% CrI: 551–710) (S-Table 5 in the Supplementary Material) consisting of 365 (95% CrI: 284–448) infected cases and 267 (95% CrI: 195–334) removed cases (S-Table 6 in the Supplementary Material). Based on this dynamic epidemic curve, 50% of passengers were infected by SARS-CoV-2 until February 22nd (S-Table 6 in the Supplementary Material). During the acceleration period of the outbreak (from Jan 10th to February 26th), there were more exposed subjects (Fig. 4b, yellow line) compared with the infected (Fig. 4b, blue line). The difference between the two components was estimated as 232 on February 19th (S-Table 6 in the Supplementary Material). The corresponding figures were 119 (51% of February 19th) and 69 (30% of Feb 19th) on Feb 14th and 11st, respectively (S-Table 6 in the Supplementary Material). This difference represents the expected number of subjects who have been exposed but not confirmed as infected cases yet would mix up with the susceptibles during evacuation. This accounted for the additional 126 confirmed cases requiring quarantine and isolation after the evacuation of those already exposed and still susceptible. The dynamic curve suggests February 26th, the crossed point between the exposed and the infected curve as shown in Fig. 4b, may be a better evacuation time of the remaining uninfected passengers and crews than the real evacuation date of February 19th.

### Heterogeneity of COVID-19 transmission on the Diamond Princess Cruise Ship by deck

S-Table 7 shows the estimated results on the transmission coefficient and R_0_ of each deck of passengers and crews. Transmission coefficients within deck were estimated as 0.72/day (95% CrI: 0.66–0.77/day) and 0.34/day (95% CrI: 0.27–0.40/day) for passengers and crews, respectively. The corresponding estimated figures of R_0_ were 5.18 and 2.46.

Among the passenger decks (level 5–14), level 12 had the highest within-deck transmission coefficient, yielding 3.22 (95% CrI: 2.84–2.57) of the estimated R_0_. It is very interesting to note that the heterogeneity of R_0_ by each deck in the estimated results of S-Table 7.

Regarding the within-deck transmission for the crews, the corresponding R_0_ figure for the major cluster of COVID-19 cases on level 3 was 2.18 (95% CrI: 1.70–2.66). S-Table 7 also lists the estimated frequency of within-deck transmission projected from the transmission coefficient of each deck.

**Fig. 5:**
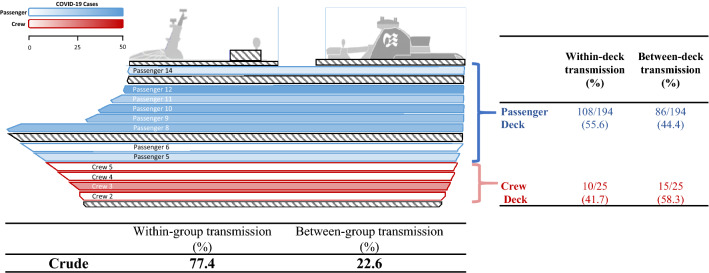
Proportion of within-deck and between-deck transmission for passengers and crews

Figure 5 shows the observed COVID-19 cases among passenger decks (blue color scale) and crew decks (red color scale). Regarding the early period of COVID-19 outbreak on the Diamond Princess Cruise Ship, the within-deck transmission (77.4%) dominated over the between-deck transmission (22.6%). When the transmission classified by passengers or crews, more than half of the cases resulted from within-deck transmission (55.6%) for the passenger group whereas the between-deck transmission made more contribution to the crew group (58.3%).

### Efficacy of containment measures

**Fig. 6:**
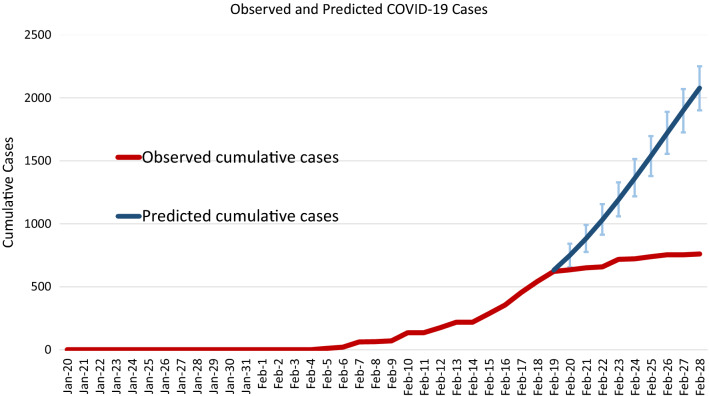
Effectiveness of containment measures on quarantine and isolation. The blue line shows the expected cumulative cases without containment measure on the Diamond Princess Cruise Ship. The red line is the observed daily cumulative case with containment measure. The total number of COVID-19 cases associated with the outbreak on the Diamond Princess Cruise Ship at February 28th was 761. Compared with the scenario of no intervention (2079.0, 95% CrI: 1901.1–2250.5) the effectiveness of quarantine and isolation in reducing the infected passenger was 63% (95% CrI: 60–66%) based on the estimate of relative risk 0.37 (95% CrI: 0.34–0.40) between the observed and the expected

Based on the estimated results on the transmissibility of SARS-CoV-2 derived from the observed data on COVID-19 outbreak, Fig. 6 shows the effectiveness of containment measures implemented on the Diamond Princess Cruise Ship. The total number of COVID-19 cases associated with the outbreak on the Diamond Princess Cruise Ship on February 28th was 761 (Fig. 6, red line). Compared with the scenario of no containment measures (2079, 95% CrI: 1901–2251, Fig. 6, blue line) the effectiveness of quarantine and partial isolation in reducing the infected passengers was 63% (95% CrI: 60–66%) based on the estimate of relative risk of 0.37 (95% CrI: 0.34–0.40) between the observed and the expected cases.

## Discussion

It is very striking to see that the basic reproductive number in such a scenario was estimated up to 5.70 (95% CrI: 4.23–7.79), which is unusually high compared with the transmissibility of SARS with the estimated R_0_ ranging from 2 to 3 (Riley et al. 2003). This unusually high value of R_0_ is reproducible in the beginning of several epidemics of COVID-19 such as Korea, Iran, and Italy and the subsequent initial epidemics waves in the Europe and in the USA. The high value of R_0_ also accounts for the soaring number of confirmed cases reported in a short period due to the accelerated transmission amplified by a series of clustered events due to public gathering. The recent outbreaks in the Washington and the New York state of USA triggered by a series of clustered events including long-term care facilities follow such a similar pattern (WHO 2020). With such a high transmissibility, the virus can lead to an overwhelming number of COVID-19 cases and further bring extraordinary stress to health care system as well as social and economic infrastructure.

Based on the simulated projection of natural dynamic epidemic curve without containment measure, the entire infectious process took 47 days from the introduction of index cases since January 20th until the end of epidemics on March 7th when 84% of passengers would be expected to be infected (3128 cases) to reach the phenomenon of herd immunity. Excluding two weeks of the incubation period from the introduction of index case, the epidemic cycle of COVID-19 on the Diamond Princess Cruise Ship is around one month. The natural dynamic curve also provides the control group for the evaluation of the effectiveness of containment measures on quarantine and isolation measures. Given 63% effectiveness of partial containment measure, had the complete containment measures been implemented on February 14th, the total number of cases would have been reduced from 761 to 403 cases, resulting in the effectiveness of 81% (95% CrI: 79-82%, S-Table 8 in the Supplementary Material) and an even larger effectiveness would have been achieved given an early implementation on February 10th suggested from the estimated dynamic curve (Fig. 4b) while the testing capacity is limited. Moreover, even when the implementation was delayed to February 14th, the measure adopted to guarantee complete containment as evacuation with adequate deposition should be a high priority. However, timely evacuation is also important as those who were arranged to be evacuated also bore a high risk due to the mixing up of subjects already exposed not yet confirmed as infected cases. The timely evacuation would be expected after the peak of the transmission dynamic on February 26th rather than the real date of February 19th. The decision on this early evacuation thus results in the subsequent occurrence of cases among these exposed subjects after evacuation returning to each country that may invoke cluster infection of public gathering and the epidemic in the community. These findings derived from the Diamond Princess can be applied to military ships. COVID-19 cases have been reported on four USA aircraft carriers since the end of March. In the outbreak occurring on the USS Theodore Roosevelt, 1271 COVID-19 cases out of 5000 onboard Navy sailors were reported (Kasper et al. 2020). Given the similar situation of clustering and close contact in a confined setting, the timely containment measures and evacuation should be implemented as suggested by the transmission dynamic derived from the Diamond Princess outbreak.

Although the daily COVID-19 cases can be derived from the proposed Bayesian SEIR model, the discrepancy between the predicted frequencies and the observed ones when only symptom-based test is adopted should be interpreted with great caution because the time scale of the empirical data in day would be fluctuated due to the accumulative cases until the presence of symptoms that is subject to incubation period. This can be clearly seen in daily reported cases of the early period from February 5th to 11th. The fluctuation has been improved until the exhaustive daily testing was provided since February 11th. Moreover, the fluctuation in daily cases due to the process of examination and reporting is obvious for February 11st and 14th, when zero cases were reported. This may due to, for example, the deployment of testing capacity (February 11st) and the evacuation of USA citizens (February 14th).

To cope with this problem, when assessing the dynamic of COVID-19 transmission on the Diamond Princess Cruise Ship, we still suggest that the parameters had better be estimated from the empirical data on the basis of cumulated cases as so doing provides the merit of smoothing the daily fluctuation due to the process of reporting COVID-19 cases particularly when the exhaustive daily testing is lacking.

Previous studies have suggested the heterogeneity in the cases on different decks and locations due to different transmission modes (Azimi et al. 2020; Fang et al. 2020; Plucinski et al. 2020; Yamagishi et al. 2020b). The fomite and aerosol transmission (van Doremalen et al. 2020; Mallapaty 2020; Mizumoto and Cowell 2020; Nakazawa et al. 2020), and meteorological factors (Cheng and Wang-Li 2019; Hsiao et al. 2020; Lin et al. 2020; Pang et al. 2020; Pani et al. 2020) have been reported to make respective contributions to the spread of SARS-CoV-2. Our results with a predominant within-deck transmission a higher likelihood for SARS-CoV-2 transmission through the aerosol transmission on this occasion that is consistent with the previous finding showing the higher likelihood of aerosol transmission as the major role of SARS-CoV-2 (Azimi et al. 2020; Liu et al. 2020). The heterogeneity of transmission coefficients in different decks may play down the role of the airborne or air-condition related transmission. For the scenario of fomite transmission, the spread of SARS-CoV-2 is mainly characterized by the contaminated surfaces in the culprit area by which a predominant between-deck transmission is expected. The higher proportion of the between-deck transmission for the crews compared with the passengers may explain the fomite transmission which was a reflection of the contact pattern through services such as the provision of beverage and food (Kakimoto et al. 2020; Liu et al. 2020; Yamagishi et al. 2020a). Similar scenarios in terms of the attack rate for crews has been reported for the Polar Expedition Cruise Ship (Ing et al. 2020).

To address the causes of spatial heterogeneity, the method of stratified spatial heterogeneity (SSH) using q statistics together with Geodetector proposed by Wang et al. (2016) can be very useful for mining the stratify heterogeneity of geography with respect to a variety of subjects including infectious disease. However, due to the limitation in the availability of data, the approach of SSH to formally assess the causes of heterogeneity cannot be performed. A future formal research by using SSH with Geodetector to elucidate the causes of heterogeneity and further to pinpoint the source of outbreak can shed light on the development of containment measures.

The SEIR model is an extension of SIR model. While the SIR model assumes no latent period, the SEIR model includes the latent period by incorporating the component of exposed (E), which captures the latency from the exposure of susceptibles to an infective (compartment I) until infectiousness. Given the substantial proportion of asymptomatic and presymptomatic cases, this extension makes the SEIR model more specific for depicting the COVID-19 outbreak on the Diamond Princess Cruise Ship. The application of compartment model enables us to depict the instantaneous change by using a series of differential equations for the dynamics of infectious disease. The transmission coefficient derived from the Bayesian SEIR model thus take into account the dynamic changes in the number of infectives, exposed, infected, and recovered. The figure of 0.75/day is a reflection of transmission coefficient. The proposed Bayesian SEIR model also provides a flexible approach to elucidate the transmission of SARS-CoV-2 by using aggregate data on observed cases with the limited resolution. Thanks to the proposed Bayesian framework and MCMC estimation, information on disease characteristics such as incubation and transmission period elicited from previous studies (Huang et al. 2020; Guan et al. 2020) can be incorporated into our models with prior distributions, making allowance for the uncertainty of parameters originated from their studies. The parameters estimated from the integration of observed data and state-of-the-art knowledge on disease pattern can shed light on the transformation of disease pattern which plays a crucial role in the allocation of resources and the implementation of strategies for us to contain the coming cruise ships that may be attacked by COVID-19.

## Supplementary information

Below is the link to the electronic supplementary material.

Supplementary file1 (DOCX 79 kb)

## Data Availability

Source data are available in the supplementary materials. The code for analysis is available from author upon request.
