# Supplementary material for: The Bayesian Susceptible-Exposed-Infected-Recovered model for the outbreak of COVID-19 on the Diamond Princess Cruise Ship
Source: Stoch Environ Res Risk Assess. 2021 Jan 26;35(7):1319–33. doi: 10.1007/s00477-020-01968-w (PMC7837082; doi:10.1007/s00477-020-01968-w)
Supplement: Supplementary file 1 — Supplementary file1 (DOCX 79 kb) [file 477_2020_1968_MOESM1_ESM.doc]

**Supplementary material**

**The Bayesian approach for modelling the dynamic of COVID-19 outbreak on the**

**Diamond Princess Cruise Ship**

Chao-Chih Lai, Chen-Yang Hsu, Hsiao-Hsuan Jen, Amy Ming-Fang Yen, Chang-Chuan Chan and

Hsiu-Hsi Chen

**A. The collection of data on COVID-19 outbreak on the Diamond Princess Cruise Ship.**

**B. Supplementary Tables**

**S-Table 1.** Cases of COVID-19, number of subjects tested and number of asymptomatic cases associated with the Diamond Princess Cruise Ship.

**S-Table 2.** The maximum capacity and population size by level of deck for passengers and crews on the Diamond Princess Cruise Ship.

**S-Table 3.** The number of fitted COVID-19 cases on the Diamond Princess cruise ship based on Bayesian SEIR model.

**S-Table 4.** Data, estimated parameters, and posterior distribution of the Bayesian SEIR model.

**S-Table 5.** The predicted number of COVID-19 cases of Diamond Princess Cruise Ship passengers up to March. 7^th^, 2020 based on estimated results derived from data up to February 19^th^, 2020 with the scenario of persistent transmission.

**S-Table 6.** Natural evolution of the compartment of susceptible (S), exposed (E), infected (I), and removed (R) predicted by Bayesian SEIR model using data on COVID-19 outbreak on the Diamond Princess Cruise Ship up to February 19^th^, 2020.

**S-Table 7.** Estimated transmission coefficient, basic reproductive number, and expected cases of within-group transmission by deck for passengers and crews.

**S-Table 8.** The predicted number of cumulative COVID-19 cases on the Diamond Princess Cruise Ship based on the scenario of complete containment measures applied on February 14^th^, 2020.

**C. Supplementary Figure**

**S-Figure 1.** Comparison between observed and fitted daily COVID-19 cases between February 12th to 19th on Diamond Princess Cruise Ship.

**A. The collection of data on COVID-19 outbreak on the Diamond Princess Cruise Ship**

The 14-day itinerary of Diamond Princess Cruise Ship was scheduled to depart from Yokohama on January 20^th^, 2020. Among the total of 3711 persons on board, there were 2666 passengers and 1045 crews. Information on the itinerary and the number of passengers of the voyage of Diamond Princess was derived from the web-based interface of the Diamond Princess Cruise Ship (WHO, 2020; Princess Cruise Lines; Ministry of Health, Labor and Welfare, Japan, 2020; Wikipedia, 2020a; Wikipedia, 2020b). The first confirmed COVID-19 case in Diamond Princess Cruise Ship was reported by Hong Kong on February 1^st^, 2020 (NIID, 2020a; Wikipedia, 2020a). Although the index case showed no symptoms during his stay in the cruise ship, he was admitted to a hospital in Hong Kong 6 days after his disembark on January 25^th^, 2020, where the infection by SARS-CoV-2 was confirmed by laboratory test. The outbreak thus started with the introduction of the index case to the susceptible passengers of the cruise ship on his embark on January 20^th^, 2020 (**Figure 2**).

The updated information on COVID-19 outbreak in Diamond Princess Cruise Ship during the quarantine period in Yokohama was provided by an official web site of Japan (2020). With information on the occurrence of the first COVID-19 case, the cruise ship was quarantined on its arrival at Yokohama on February 3^rd^, 2020. Among the 31 passengers test for SARS-CoV-2 on February 5^th^, 2020, 10 positive results were reported by the Ministry of Health, Labor and Welfare, Japan. The updated information on the number of test performed and positive cases were announced on the website of Ministry of Health, Labor and Welfare, Japan since February 5^th^ on daily basis (Wikipedia, 2020a). The number of asymptomatic passengers with positive tests was reported since February 15^th^, 2020. **S-Table 1** in the Supplementary material lists the reported count retrieved from the web-based situation report from the Ministry of Health, Labor and Welfare, Japan (2020) with the numbers cross-checked with reports and line list summarized in medias and the Princess Cruise website (WHO, 2020; Princess Cruise Lines, 2020; Wikipedia, 2020a).

The quarantine procedures implemented in the Diamond Princess Cruise Ship including the isolation of passengers and crews in cabins and the surveillance for fever and respiratory symptoms that provide the rationale for testing SARS-CoV-2 infection (NIID, 2020a). Passengers were required to stay in their cabinet to reduce the contacts and social activities during the period of quarantine. However, the strict quarantine procedures were difficult to be realized thoroughly in the cruise ship with the maintenance of operation such as dinning and services provided by crews (NIID, 2020a; NIID, 2020b; Princess Cruise Lines, 2020). With the expansion in the capacity for testing SARS-CoV-2, the Diamond Princess Cruise Ship passengers and crews were tested systematically since February 14^th^. The enhanced measures for isolation policy in the contemporaneous period were also started. A 14-day quarantine period (February 5^th^ to February 19^th^) was predetermined by the Ministry of Health, Labor and Welfare, Japan in the light of when passengers and crews free of symptoms and contact with confirmed cases were dismissed form the quarantine status (Ministry of Health, Labor and Welfare, Japan, 2020).

**B. Supplementary Tables**

**S-Table 1. Cases of COVID-19, number of subjects tested and number of asymptomatic cases associated with the Diamond Princess Cruise Ship.**

| **Date** | **Subjects Tested,**  **JP** | **Total Cases**  **, JP** | **Total Cases**  **Outside JP** | **Asymptomatic Cases, JP** | **Positive rate,**  **JP** | **Asymptomatic Proportion, JP** |
| --- | --- | --- | --- | --- | --- | --- |
| **Jan-20** | 0 | 1 | - | - | - | - |
| **Jan-21** | 0 | 0 | - | - | - | - |
| **Jan-22** | 0 | 0 | - | - | - | - |
| **Jan-23** | 0 | 0 | - | - | - | - |
| **Jan-24** | 0 | 0 | - | - | - | - |
| **Jan-25** | 0 | 0 | - | - | - | - |
| **Jan-26** | 0 | 0 | - | - | - | - |
| **Jan-27** | 0 | 0 | - | - | - | - |
| **Jan-28** | 0 | 0 | - | - | - | - |
| **Jan-29** | 0 | 0 | - | - | - | - |
| **Jan-30** | 0 | 0 | - | - | - | - |
| **Jan-31** | 0 | 0 | - | - | - | - |
| **Feb-1** | 0 | 0 | - | - | - | - |
| **Feb-2** | 0 | 0 | - | - | - | - |
| **Feb-3** | 0 | 0 | - | - | - | - |
| **Feb-4** | 0 | 0 | - | - | - | - |
| **Feb-5** | 31 | 10 | - | - | 32.3 | - |
| **Feb-6** | 71 | 10 | - | - | 14.1 | - |
| **Feb-7** | 171 | 41 | - | - | 24.0 | - |
| **Feb-8** | 6 | 3 | - | - | 50.0 | - |
| **Feb-9** | 57 | 6 | - | - | 10.5 | - |
| **Feb-10** | 103 | 65 | - | - | 63.1 | - |
| **Feb-11** | 0 | 0 | - | - | - | - |
| **Feb-12** | 53 | 39 | - | - | 73.6 | - |
| **Feb-13** | 221 | 44 | - | - | 19.9 | - |
| **Feb-14** | 0 | 0 | - | - | - | - |
| **Feb-15** | 217 | 67 | - | 73* | 30.9 | 25.5 |
| **Feb-16** | 289 | 70 | - | 38 | 24.2 | 54.3 |
| **Feb-17** | 504 | 99 | - | 78 | 19.6 | 78.8 |
| **Feb-18** | 681 | 74 | 14 (USA) | 65 | 10.9 | 87.8 |
| **Feb-19** | 607 | 79 | 0 | 68 | 13.0 | 86.1 |
| **Feb-20** | - | 13 | 0 | - | - | - |
| **Feb-21** | - | 14 | 2 (AU) | - | - | - |
| **Feb-22** | - | 0 | 4 (AU) | - | - | - |
|  |  |  | 4 (USA) |  |  |  |
| **Feb-23** | - | 57 | 1 (AU) | - | - | - |
|  |  |  | 1 (IS) |  |  |  |
| **Feb-24** | - | 0 | 4 (UK) | - | - | - |
| **Feb-25** | - | 0 | 18 (USA) | - | - | - |
| **Feb-26** | - | 14 | 0 | - | - | - |
| **Feb-27** | - | 0 | 0 | - | - | - |
| **Feb-28** | - | 0 | 1 (HK) | - | - | - |
|  |  |  | 6 (USA) |  |  |  |
| **Overall** | **3011** | **706** | **55** | **322** | **20.2** | **51.8** |

Abbreviations: AU: Australia, HK: Hong Kong, IS: Israel, JP: Japan, UK: United Kingdom, USA: United States of America; JP represents the procedures/cases reported in Japan

*Asymptomatic cases identified between February 5^th^ to February 15^th^, 2020.

**S-Table 2. The maximum capacity, population size, and COVID-19 cases by decks for passengers and crews on the Diamond Princess Cruise Ship***

1. **Passenger deck**

| **Deck** | **Maximum capacity** | **Passengers onboard** | **COVID-19 cases (%) up to Feb-13** |
| --- | --- | --- | --- |
| **Passenger Level 14** | 138 | 133 | 10 (7.7) |
| **Passenger Level 12** | 547 | 467 | 45 (9.5) |
| **Passenger Level 11** | 657 | 540 | 24 (4.5) |
| **Passenger Level 10** | 644 | 525 | 37 (7.0) |
| **Passenger Level 9** | 640 | 438 | 31 (7.0) |
| **Passenger Level 8** | 471 | 376 | 33 (8.8) |
| **Passenger Level 6** | 28 | 27 | 0 (0) |
| **Passenger Level 5** | 192 | 159 | 14 (8.8) |
| **Overall** | 3317 | 2666 | 194 (7.3) |

1. **Crew deck**

| **Deck** | **Crews**  **onboard** | **COVID-19 cases (%)**  **up to Feb-13** |
| --- | --- | --- |
| **Crew Level 14** | 12 | 0 (0) |
| **Crew Level 12** | 6 | 0 (0) |
| **Crew Level 7** | 29 | 0 (0) |
| **Crew Level 6** | 32 | 0 (0) |
| **Crew Level 5** | 82 | 1 (1.5) |
| **Crew Level 4** | 145 | 1 (0.9) |
| **Crew Level 3** | 571 | 20 (3.6) |
| **Crew Level 2** | 168 | 3 (1.5) |
| **Overall** | 1045 | 25 (2.4) |

*****The frequencies of passengers, crews, and cases of each deck were derived from the proportion reported by Yamagishi et al. (2020(a)).

**S-Table 3. The number of fitted COVID-19 cases on the Diamond Princess Cruise Ship based on the Bayesian SEIR model.**

| **Date** | **Fitted Daily Frequencies** | | | **Observed Cases** |
| --- | --- | --- | --- | --- |
|  | **Infected** | | |  |
|  | **Mean** | **95% CrI** | |  |
| **Feb-5** | 7.0 | 6.3 | 7.8 | 10 |
| **Feb-6** | 8.7 | 7.8 | 9.7 | 10 |
| **Feb-7** | 10.8 | 9.6 | 12.0 | 41 |
| **Feb-8** | 13.4 | 11.8 | 14.9 | 3 |
| **Feb-9** | 16.5 | 14.4 | 18.4 | 6 |
| **Feb-10** | 20.3 | 17.8 | 22.9 | 65 |
| **Feb-11** | 24.9 | 21.6 | 28.0 | 0 |
| **Feb-12** | 30.5 | 26.4 | 34.4 | 39 |
| **Feb-13** | 37.1 | 32.1 | 41.9 | 44 |
| **Feb-14** | 44.9 | 38.8 | 50.9 | 0 |
| **Feb-15** | 54.1 | 46.5 | 61.1 | 67 |
| **Feb-16** | 64.6 | 56.0 | 73.3 | 70 |
| **Feb-17** | 76.6 | 66.3 | 86.6 | 99 |
| **Feb-18** | 89.7 | 78.4 | 101.5 | 88 |
| **Feb-19** | 103.9 | 91.3 | 117.1 | 79 |

**S-Table 4. Data, estimated parameters, and posterior distribution of the Bayesian SEIR model.**

| **Parameters** | | **Prior distribution** | **Information Sources** | **Variable** | | **Posterior distribution** | |
| --- | --- | --- | --- | --- | --- | --- | --- |
| β | Overall transmission coefficient | Gamma (0.001, 0.001) | Estimated from empirical data  (Supplementary Material A,  S-Table 1 in Supplementary Material) | | Count of cases and suspectibles on the Diamond Princess Cruise Ship |  | Gamma (6087,7725) |
| log(β*_jj_*) | Transmission coefficient by deck (log transformation) | Normal (0, 10^4^) | Estimated from empirical data  (S-Table 2 in Supplementary Material) | | Count of cases and suspectibles by deck and personnel of the Diamond Princess Cruise Ship | Passenger | Normal (-0.03, 6.7×10^-4^) |
|  |  |  |  |  |  | Crew | Normal (-0.78, 8.9×10^-3^) |
|  |  |  |  |  |  | Deck 5 | Normal (-1.12, 3.2×10^-2^) |
|  |  |  |  |  |  | Deck 8 | Normal (-0.64, 5.6×10^-3^) |
|  |  |  |  |  |  | Deck 9 | Normal (-0.67, 6.2×10^-3^) |
|  |  |  |  |  |  | Deck 10 | Normal (-0.59, 4.7×10^-3^) |
|  |  |  |  |  |  | Deck 11 | Normal (-0.80, 9.5×10^-3^) |
|  |  |  |  |  |  | Deck 12 | Normal (-0.51, 3.5×10^-3^) |
|  |  |  |  |  |  | Deck 14 | Normal (-1.43, 1.3×10^-1^) |
|  |  |  |  |  |  | Deck 3 | Normal (-0.90, 1.3×10^-2^) |
| σ | Inverse of  average incubation period | Gamma (53, 278) | Guan et al., 2020 | | Median and IQR |  | Gamma (5170, 27339) |
| α | Average rate of  recovery or removal | Gamma(24, 168) | Huang et al., 2020 | | Median and IQR |  | Gamma (2355, 16564) |

**S-Table 5. The predicted number of COVID-19 cases of Diamond Princess Cruise Ship passengers up to March 7^th^, 2020 based on the estimated results derived from data up to February 19^th^, 2020 with the scenario of persistent transmission by using the Bayesian SEIR model.**

| **Date** | **Observed Cumulated Cases^*^** | **Fitted/Predicted Cases** | | |
| --- | --- | --- | --- | --- |
|  |  | **Estimate** | **95% Credible Interval** | |
| **Feb-5** | 11 | 35.9 | 32.5 | 39.0 |
| **Feb-6** | 21 | 44.6 | 40.2 | 48.6 |
| **Feb-7** | 62 | 55.4 | 50.0 | 60.8 |
| **Feb-8** | 65 | 68.8 | 61.8 | 75.6 |
| **Feb-9** | 71 | 85.3 | 76.5 | 94.3 |
| **Feb-10** | 136 | 105.5 | 94.3 | 117.2 |
| **Feb-11** | 136 | 130.4 | 116.0 | 145.1 |
| **Feb-12** | 175 | 160.9 | 142.0 | 179.0 |
| **Feb-13** | 219 | 198.0 | 174.8 | 221.5 |
| **Feb-14** | 219 | 242.9 | 213.8 | 272.6 |
| **Feb-15** | 286 | 297.0 | 258.7 | 332.1 |
| **Feb-16** | 356 | 361.7 | 314.9 | 405.4 |
| **Feb-17** | 455 | 438.2 | 383.0 | 493.6 |
| **Feb-18** | 543 | 527.9 | 459.9 | 593.0 |
| **Feb-19** | 622 | 631.9 | 551.0 | 709.9 |
| **Feb-20** | 635 | 750.5 | 655.6 | 841.9 |
| **Feb-21** | 651 | 884.0 | 776.4 | 991.2 |
| **Feb-22** | 659 | 1031.5 | 913.7 | 1157.1 |
| **Feb-23** | 718 | 1191.5 | 1059.4 | 1329.8 |
| **Feb-24** | 722 | 1361.7 | 1218.8 | 1514.8 |
| **Feb-25** | 740 | 1539.1 | 1380.0 | 1696.8 |
| **Feb-26** | 754 | 1720.3 | 1555.4 | 1889.1 |
| **Feb-27** | 754 | 1901.4 | 1726.0 | 2070.3 |
| **Feb-28** | 761 | 2079.0 | 1901.1 | 2250.5 |
| **Feb-29** | - | 2250.0 | 2073.0 | 2422.1 |
| **Mar-1** | - | 2411.8 | 2239.0 | 2583.0 |
| **Mar-2** | - | 2562.4 | 2390.8 | 2726.9 |
| **Mar-3** | - | 2700.8 | 2539.6 | 2864.9 |
| **Mar-4** | - | 2826.4 | 2667.6 | 2980.5 |
| **Mar-5** | - | 2939.2 | 2790.4 | 3089.2 |
| **Mar-6** | - | 3039.5 | 2898.2 | 3180.4 |
| **Mar-7** | - | 3128.1 | 2995.2 | 3261.1 |

**^*^**Cumulated cases include one index case, cases confirmed in Japan and outside Japan.

**S-Table 6. Natural evolution of each compartment predicted by the Bayesian SEIR model using data on COVID-19 outbreak on the Diamond Princess Cruise Ship up to February 19^th^, 2020.**

| **Date** | **Susceptible** | | | **Exposed** | | | **Infected** | | | **Removed** | | |
| --- | --- | --- | --- | --- | --- | --- | --- | --- | --- | --- | --- | --- |
|  | **Estimate** | **95% CrI** | | **Estimate** | **95% CrI** | | **Estimate** | **95% CrI** | | **Estimate** | **95% CrI** | |
| **Jan-20** | 3709.3 | 3709.1 | 3709.4 | 0.7 | 0.6 | 0.8 | 0.9 | 0.9 | 1.0 | 0.1 | 0.1 | 0.2 |
| **Jan-21** | 3708.5 | 3708.2 | 3708.8 | 1.3 | 1.0 | 1.6 | 1.0 | 0.9 | 1.1 | 0.3 | 0.2 | 0.4 |
| **Jan-22** | 3707.7 | 3707.3 | 3708.0 | 1.8 | 1.4 | 2.2 | 1.1 | 1.0 | 1.2 | 0.4 | 0.3 | 0.6 |
| **Jan-23** | 3706.7 | 3706.2 | 3707.2 | 2.4 | 1.8 | 2.9 | 1.3 | 1.2 | 1.5 | 0.6 | 0.4 | 0.8 |
| **Jan-24** | 3705.6 | 3704.9 | 3706.2 | 3.0 | 2.3 | 3.7 | 1.6 | 1.5 | 1.8 | 0.8 | 0.5 | 1.1 |
| **Jan-25** | 3704.1 | 3703.3 | 3705.0 | 3.8 | 2.9 | 4.7 | 2.0 | 1.8 | 2.2 | 1.1 | 0.7 | 1.4 |
| **Jan-26** | 3702.4 | 3701.3 | 3703.5 | 4.8 | 3.7 | 5.9 | 2.5 | 2.2 | 2.7 | 1.4 | 0.9 | 1.8 |
| **Jan-27** | 3700.2 | 3698.8 | 3701.5 | 5.9 | 4.6 | 7.4 | 3.1 | 2.8 | 3.4 | 1.8 | 1.2 | 2.3 |
| **Jan-28** | 3697.5 | 3695.7 | 3699.3 | 7.4 | 5.7 | 9.3 | 3.8 | 3.4 | 4.3 | 2.3 | 1.5 | 2.9 |
| **Jan-29** | 3694.1 | 3691.9 | 3696.4 | 9.2 | 7.1 | 11.5 | 4.8 | 4.2 | 5.3 | 2.9 | 2.0 | 3.7 |
| **Jan-30** | 3689.9 | 3686.9 | 3692.7 | 11.5 | 8.7 | 14.4 | 6.0 | 5.2 | 6.7 | 3.6 | 2.5 | 4.7 |
| **Jan-31** | 3684.7 | 3680.9 | 3688.3 | 14.3 | 10.9 | 18.0 | 7.4 | 6.5 | 8.3 | 4.6 | 3.1 | 5.9 |
| **Feb-1** | 3678.3 | 3673.4 | 3682.8 | 17.8 | 13.5 | 22.5 | 9.2 | 8.1 | 10.5 | 5.7 | 3.9 | 7.4 |
| **Feb-2** | 3670.2 | 3664.1 | 3676.1 | 22.1 | 16.5 | 27.8 | 11.5 | 10.1 | 13.1 | 7.2 | 5.0 | 9.3 |
| **Feb-3** | 3660.2 | 3651.8 | 3667.1 | 27.5 | 20.4 | 34.6 | 14.3 | 12.4 | 16.4 | 9.0 | 6.3 | 11.6 |
| **Feb-4** | 3647.8 | 3637.2 | 3656.7 | 34.2 | 25.3 | 43.2 | 17.8 | 15.4 | 20.5 | 11.2 | 7.9 | 14.6 |
| **Feb-5** | 3632.5 | 3619.3 | 3644.0 | 42.4 | 31.3 | 53.8 | 22.1 | 19.0 | 25.6 | 14.0 | 9.9 | 18.2 |
| **Feb-6** | 3613.6 | 3597.2 | 3628.5 | 52.6 | 38.5 | 66.6 | 27.4 | 23.4 | 31.9 | 17.5 | 12.5 | 22.7 |
| **Feb-7** | 3590.2 | 3569.4 | 3609.1 | 65.0 | 47.6 | 82.9 | 34.0 | 28.8 | 39.7 | 21.8 | 15.6 | 28.3 |
| **Feb-8** | 3561.4 | 3535.1 | 3585.5 | 80.4 | 58.1 | 102.2 | 42.2 | 35.4 | 49.4 | 27.1 | 19.3 | 35.1 |
| **Feb-9** | 3526.0 | 3494.3 | 3557.7 | 99.1 | 71.3 | 126.3 | 52.2 | 43.7 | 61.5 | 33.8 | 24.1 | 43.7 |
| **Feb-10** | 3482.7 | 3443.4 | 3522.9 | 121.8 | 88.4 | 156.7 | 64.5 | 53.7 | 76.5 | 41.9 | 30.1 | 54.3 |
| **Feb-11** | 3430.1 | 3381.0 | 3480.8 | 149.3 | 110.8 | 195.1 | 79.6 | 65.8 | 94.8 | 52.0 | 38.0 | 67.9 |
| **Feb-12** | 3366.3 | 3305.0 | 3429.0 | 182.3 | 135.1 | 238.6 | 97.9 | 80.5 | 117.3 | 64.4 | 46.9 | 83.7 |
| **Feb-13** | 3289.6 | 3213.2 | 3366.2 | 221.5 | 163.8 | 290.3 | 120.1 | 98.0 | 144.4 | 79.7 | 58.0 | 103.2 |
| **Feb-14** | 3198.1 | 3104.0 | 3292.1 | 267.7 | 192.4 | 345.8 | 146.9 | 119.2 | 177.3 | 98.4 | 71.5 | 126.9 |
| **Feb-15** | 3090.0 | 2967.3 | 3195.1 | 321.1 | 236.9 | 420.8 | 178.8 | 144.1 | 216.8 | 121.2 | 89.0 | 156.9 |
| **Feb-16** | 2963.7 | 2821.3 | 3093.1 | 382.1 | 272.6 | 490.4 | 216.5 | 173.0 | 263.1 | 148.8 | 109.6 | 192.3 |
| **Feb-17** | 2818.2 | 2652.1 | 2971.1 | 450.2 | 329.5 | 584.1 | 260.5 | 207.3 | 318.3 | 182.1 | 134.4 | 234.5 |
| **Feb-18** | 2653.3 | 2459.4 | 2830.2 | 524.5 | 387.1 | 680.1 | 311.2 | 244.7 | 379.8 | 222.1 | 164.2 | 284.9 |
| **Feb-19** | 2469.9 | 2250.9 | 2672.8 | 603.0 | 443.1 | 774.8 | 368.6 | 289.4 | 452.6 | 269.6 | 199.5 | 344.1 |
| **Feb-20** | 2270.1 | 2034.8 | 2506.5 | 683.0 | 502.8 | 870.8 | 432.4 | 340.8 | 536.0 | 325.5 | 241.7 | 413.4 |
| **Feb-21** | 2057.6 | 1791.9 | 2306.2 | 760.9 | 566.6 | 965.1 | 501.8 | 395.9 | 626.7 | 390.7 | 291.6 | 493.8 |
| **Feb-22** | 1837.5 | 1563.6 | 2109.4 | 832.5 | 625.1 | 1047.6 | 575.2 | 440.0 | 708.3 | 465.9 | 350.5 | 586.5 |
| **Feb-23** | 1615.7 | 1334.7 | 1899.9 | 893.2 | 670.6 | 1108.7 | 650.8 | 497.6 | 806.3 | 551.3 | 414.2 | 686.9 |
| **Feb-24** | 1398.7 | 1125.3 | 1694.0 | 939.2 | 711.4 | 1155.5 | 725.9 | 554.1 | 905.7 | 647.3 | 489.7 | 800.9 |
| **Feb-25** | 1192.6 | 927.4 | 1485.8 | 967.2 | 755.7 | 1197.6 | 797.9 | 610.7 | 1007.8 | 753.4 | 573.7 | 925.1 |
| **Feb-26** | 1002.6 | 744.3 | 1281.5 | 975.6 | 769.8 | 1199.8 | 863.8 | 660.5 | 1102.0 | 869.0 | 676.7 | 1068.6 |
| **Feb-27** | 832.4 | 587.1 | 1089.1 | 964.5 | 760.9 | 1177.6 | 921.1 | 690.6 | 1174.6 | 993.1 | 780.5 | 1210.8 |
| **Feb-28** | 683.9 | 466.1 | 926.3 | 935.4 | 735.0 | 1134.5 | 967.5 | 716.7 | 1242.0 | 1124.3 | 890.6 | 1357.3 |
| **Feb-29** | 557.5 | 354.5 | 769.7 | 891.1 | 695.6 | 1080.1 | 1001.6 | 727.3 | 1290.7 | 1260.8 | 1007.2 | 1509.7 |
| **Mar-1** | 452.1 | 274.6 | 646.7 | 835.3 | 659.2 | 1028.4 | 1022.6 | 719.9 | 1319.1 | 1401.0 | 1127.2 | 1662.1 |
| **Mar-2** | 365.7 | 205.7 | 534.3 | 771.6 | 609.4 | 959.6 | 1030.6 | 724.2 | 1353.4 | 1543.1 | 1254.0 | 1816.4 |
| **Mar-3** | 296.0 | 150.9 | 439.9 | 703.7 | 547.8 | 881.5 | 1026.2 | 691.4 | 1346.8 | 1685.1 | 1381.2 | 1967.6 |
| **Mar-4** | 240.2 | 116.6 | 369.9 | 634.8 | 484.7 | 800.9 | 1010.5 | 676.3 | 1351.2 | 1825.5 | 1509.5 | 2115.6 |
| **Mar-5** | 195.9 | 86.9 | 308.8 | 567.1 | 424.0 | 721.5 | 985.1 | 648.7 | 1334.9 | 1962.9 | 1655.3 | 2273.8 |
| **Mar-6** | 161.0 | 67.7 | 262.5 | 502.6 | 368.4 | 649.7 | 951.5 | 613.3 | 1304.7 | 2096.0 | 1788.8 | 2413.5 |
| **Mar-7** | 133.4 | 52.8 | 224.7 | 442.4 | 320.0 | 583.0 | 911.5 | 578.5 | 1271.8 | 2223.7 | 1906.6 | 2533.2 |

**S-Table 7. Estimated transmission coefficient, basic reproductive number, and expected cases of within-deck transmission by deck for passengers and crews up to Feb 13^th^, 2020.**

| **Deck** | | **Transmission coefficient (**β*_jj_***)** | | | | **Basic reproductive number (**R_0_**)** | | | | **Estimated number of within-deck transmitted**  **COVID-19 cases** |
| --- | --- | --- | --- | --- | --- | --- | --- | --- | --- | --- |
|  |  | **Estimate** | | **95% CrI** | | **Estimate** | | **95% CrI** | |  |
| **Passenger** | **14** | 0.19 | 0.09 | | 0.28 | 1.34 | 0.64 | | 2.02 | 4.1 |
|  | **12** | 0.45 | 0.40 | | 0.50 | 3.22 | 2.84 | | 3.57 | 27.5 |
|  | **11** | 0.34 | 0.28 | | 0.40 | 2.41 | 2.01 | | 2.90 | 13.0 |
|  | **10** | 0.41 | 0.36 | | 0.47 | 2.95 | 2.57 | | 3.33 | 21.2 |
|  | **9** | 0.38 | 0.33 | | 0.44 | 2.75 | 2.34 | | 3.18 | 17.2 |
|  | **8** | 0.39 | 0.33 | | 0.45 | 2.82 | 2.42 | | 3.22 | 18.3 |
|  | **5** | 0.25 | 0.16 | | 0.32 | 1.77 | 1.19 | | 2.33 | 6.5 |
|  | **Crude** | 0.72 | 0.66 | | 0.77 | 5.18 | 4.93 | | 5.44 | 156.4 |
| **Crew** | **3** | 0.30 | 0.24 | | 0.37 | 2.18 | 1.70, | | 2.66 | 9.7 |
|  | **Crude** | 0.34 | 0.27 | | 0.40 | 2.46 | 2.00 | | 2.88 | 13.1 |
| **All onboard subjects** | | 0.79 | 0.62 | | 1.02 | 5.67 | 4.09 | | 8.02 | 234.4 |

**S-Table 8. The predicted number of cumulative COVID-19 cases on the Diamond Princess Cruise Ship based on the scenario of complete containment measures applied on February 14^th^, 2020.**

| **Date** | **Observed Cumulated Cases*** | **Predicted Cases** | | |
| --- | --- | --- | --- | --- |
|  |  | **Estimate** | **95% CrI** | |
| **Feb-5** | 11 | 35.9 | 32.4 | 39.1 |
| **Feb-6** | 21 | 44.6 | 40.4 | 49.0 |
| **Feb-7** | 62 | 55.4 | 49.7 | 60.8 |
| **Feb-8** | 65 | 68.8 | 61.6 | 75.8 |
| **Feb-9** | 71 | 85.3 | 76.3 | 94.6 |
| **Feb-10** | 136 | 105.6 | 94.4 | 117.9 |
| **Feb-11** | 136 | 130.5 | 116.5 | 146.6 |
| **Feb-12** | 175 | 160.9 | 142.5 | 180.7 |
| **Feb-13** | 219 | 198.0 | 175.6 | 223.9 |
| **Feb-14** | 219 | 235.2 | 205.9 | 264.5 |
| **Feb-15** | 286 | 266.0 | 232.9 | 299.9 |
| **Feb-16** | 356 | 291.5 | 253.5 | 328.1 |
| **Feb-17** | 455 | 312.6 | 271.7 | 353.1 |
| **Feb-18** | 543 | 330.1 | 288.4 | 377.0 |
| **Feb-19** | 622 | 344.6 | 297.8 | 392.8 |
| **Feb-20** | 635 | 356.7 | 304.9 | 406.0 |
| **Feb-21** | 651 | 366.7 | 313.0 | 420.1 |
| **Feb-22** | 659 | 375.1 | 319.9 | 433.3 |
| **Feb-23** | 718 | 382.0 | 323.7 | 442.8 |
| **Feb-24** | 722 | 387.8 | 326.5 | 450.5 |
| **Feb-25** | 740 | 392.6 | 330.1 | 458.6 |
| **Feb-26** | 754 | 396.6 | 330.5 | 462.7 |
| **Feb-27** | 754 | 400.0 | 331.9 | 467.4 |
| **Feb-28** | 761 | 402.8 | 334.3 | 473.7 |

**^*^**Cumulated cases include one index case, cases confirmed in Japan and outside Japan.

**S-Figure 1**. **Comparison between observed and fitted daily COVID-19 cases between February 12^th^ to 19^th^ on Diamond Princess Cruise Ship.**
